# Un kyste hydatique mimant une tumeur rénale

**DOI:** 10.11604/pamj.2019.33.206.17138

**Published:** 2019-07-15

**Authors:** Soufiane Ennaciri, Halim Lababidi

**Affiliations:** 1 Service d'Urologie, Centre Hospitalier Universitaire Hassan II, Fès, Maroc; 2 Service d'Urologie, Centre Hospitalier Gaston Ramon, Sens, France

**Keywords:** Kyste hydatique, rein, tumeur rénale, Hydatid cyst, kidney, kidney cancer

## Abstract

Hydatid cyst of the kidney is a rare condition caused by visceral hydatidoses, accounting for 2-4% of all disease locations. It is due to the larval stage of taenia of dogs, the echinococcus granulosus, which is a real public health problem in some of the Mediterranean countries. Pseudo-tumoral type IV hydatid cyst mainly poses diagnostic problems because it can mimick a neoplasm. We here report the case of a 54-year old patient with no previous medical history, in whom right tumoral mass of the kidney was discovered fortuitously on angioscanner performed due to arteritis of lower limbs. Thoraco-abdominopelvic scan showed heterogeneous expansive mid renal and upper renal pole process measuring 50mm in diameter, with calcifications, partially contrast ehanced, without secondary locations. The patient was completely asymptomatic, he didn't have hydaturia or other urinary signs. After multidisciplinary consultation, total right enlarged nephrectomy was performed and anatomo-pathological examination showed hydatid cyst of the kidney without signs of malignancy.

## Image en médecine

Le kyste hydatique du rein constitue une atteinte rare des hydatidoses viscérales, estimée entre 2 et 4% de l'ensemble des localisations de la maladie. Il est dû à la forme larvaire d'un Tænia du chien, l'echinococcus granulosus, véritable problème de santé publique dans certains pays de la Méditerranée. Le kyste hydatique pseudo tumoral, stade IV pose particulièrement un problème diagnostique en mimant une pathologie néoplasique. Nous rapportons le cas d'un patient de 54 ans, sans antécédents particuliers, chez qui on a découvert fortuitement lors d'un angioscanner réalisé dans le cadre d'une artérite des membres inférieurs, une masse rénale droite d'allure tumorale. Le scanner thoraco-abdomino-pelvien décrit un processus expansif médio rénal et polaire supérieur de 50mm de diamètre, hétérogène avec des calcifications et prenant partiellement le contraste sans localisation secondaire par ailleurs. Le patient était complétement asymptomatique, il n'avait pas d'hydaturie ni d'autres signes urinaires. Après réunion de concertation pluridisciplinaire, une néphrectomie totale élargie droite a été réalisée et l'examen anatomopathologique a montré qu'il s'agissait d'un kyste hydatique du rein sans signes de malignité.

**Figure 1:**
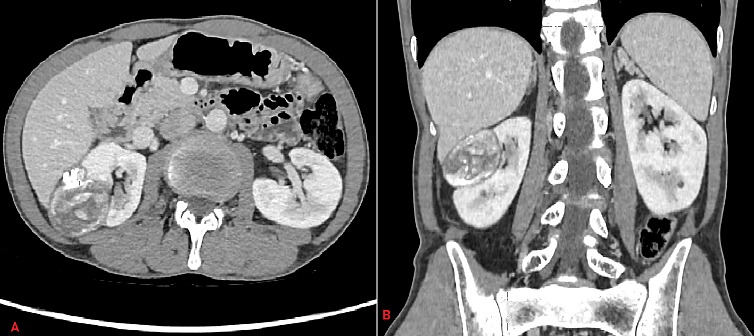
A) coupes scanographiques abdominales axial; B) et frontal, avec injection de produit de contraste, qui montrent un kyste hydatique rénal pseudo-tumoral droit avec son aspect hétérogène avec des calcifications par endroit, mimant ainsi une tumeur rénale

